# Comparison of the effects of nursing and peer education on quality of life and resilience in patients with multiple sclerosis: A randomized clinical trial

**DOI:** 10.1016/j.heliyon.2024.e39453

**Published:** 2024-10-16

**Authors:** Mohammad Mehdi Siahvashi, Morteza Shamsizadeh, Leli Tapak, Masoud Ghiasian, Azim Azizi

**Affiliations:** a Department of Medical Surgical Nursing, School of Nursing and Midwifery, Student Research Committee, Hamadan University of Medical Sciences, Hamadan, Iran; b Department of Medical Surgical Nursing, School of Nursing and Midwifery, Chronic Diseases (Home Care) Research Center, Hamadan University of Medical Sciences, Hamadan, Iran; c Department of Biostatistics, School of Public Health, Modeling of Noncommunicable Diseases Research Center, Hamadan University of Medical Sciences, Hamadan, Iran; d Department of Neuroimmunology, Faculty of Medicine, Hamadan University of Medical Sciences, Hamadan, Iran

**Keywords:** Nurses, Peer group, Resilience, Quality of life, Multiple sclerosis, Education

## Abstract

**Background:**

Multiple sclerosis (MS) is a prevalent disease of the central nervous system that often leads to diminished resilience and quality of life in affected individuals. This study aims to compare the effects of nursing and peer education on improving the quality of life and resilience in patients with multiple sclerosis.

**Materials and methods:**

This three-group clinical trial study was conducted before and after interventions at the MS Society in Hamedan, Iran. In this study, 120 patients with MS were randomly selected and assigned to three groups: nurse education, peer education, and control. Quality of life and resilience questionnaires were self-reported before and two months after the completion of the intervention. The educational sessions were conducted over five meetings, each lasting between 45 and 60 min, held every two days. Group one received education led by nurses, while group two participated in peer-led education sessions.

**Results:**

The results indicated that before the intervention, there were no significant statistical differences in demographic variables, resilience, and quality of life among the three groups (p > 0.05). However, after the intervention, both the peer and nurse groups showed a significant increase in quality of life and resilience compared to the control group (p < 0.05). Nevertheless, no differences were observed between the nurse and peer groups regarding these variables (p > 0.05).

**Conclusions:**

Both nursing and peer education methods had an equal impact on enhancing the resilience and quality of life of patients with MS. It is recommended that peer support be utilized to empower MS patients, especially in contexts where there is a shortage of nurses.

## 1 Introduction

Multiple sclerosis (MS) is a prevalent disease of the central nervous system, characterized by inflammation and the destruction of the myelin sheath [1]. It affects approximately 2.8 million people globally (35.9 per 100,000 individuals) and 95,000 individuals in Iran (115.94 per 100,000 individuals) [2].

Patients with MS often encounter numerous challenges that can have a negative impact on their quality of life. Factors such as disability, exacerbated symptoms, prolonged disease duration, stress, anxiety, depression, lack of social support, social isolation, unemployment, reduced self-care, pain, muscle spasticity, fatigue, urinary incontinence, cognitive impairments, sleep disorders, adverse effects of treatment, poor disease prognosis, and unpredictable disease progression significantly diminish the quality of life for individuals with MS [[3], [4], [5], [6]].

MS can result in physical disabilities, cognitive impairments, and mood changes, leading to decreased social support and, consequently, reduced resilience among patients. The disease also undermines one's sense of autonomy and control over their life, affecting personal resilience. Therefore, enhancing resilience in MS patients is crucial and necessitates comprehensive educational and supportive strategies to effectively address the challenges posed by the disease [5,7]. Developing and maintaining resilience in the face of chronic illness can contribute to improved physical health, better management of psychiatric symptoms, reduced disability, and an enhanced quality of life [8].

To enhance the quality of life and resilience of MS patients, it is crucial to implement appropriate nursing interventions that address these influential factors [9]. Various methods have been employed to improve these patients' quality of life and resilience, including self-compassion [10], physical exercise programs [11], informational support [12], and counseling [13], all delivered by experts, with nurses playing a pivotal role in patient education.

Education is fundamental to rehabilitation and maintaining patient health, constituting a core professional duty for nurses [14]. However, the short hospital stays of MS patients, hospitalization during acute phases of the disease, decreased receptivity to education during these times, a nursing shortage in healthcare, rehabilitation, and MS association centers, brief medical consultations, and inadequate education provision suggest compromised quality of life and resilience among MS patients [4,15]. Therefore, alternative methods such as MS specialist teams, MS nurses, or peer educators should be employed to educate these patients in specialized clinics or MS associations [16].

Peer education is a well-established instructional method that enhances health outcomes and creates an optimal learning environment [17]. This form of education is delivered by individuals knowledgeable about and actively managing the disease. Through this educational approach, the shared characteristics of group members promote empathy, social identity, and stronger connections, enabling them to share experiences and encourage each other to adopt healthy behaviors [6].

Peer education is a widely recognized instructional method that leverages the experiences and knowledge of individuals who share similar characteristics or experiences with the target group. One of the foundational theories underpinning peer education is Bandura's Social Learning Theory. This theory posits that people learn from one another through observation, imitation, and modeling. According to Bandura, learning is a cognitive process that takes place in a social context and can occur purely through observation or direct instruction, even in the absence of motor reproduction or direct reinforcement [18,19].

There is limited research on the impact of peer education on the outcomes of patients with multiple sclerosis (MS), with most studies being either two-group or single-group designs, yielding contradictory results [6,[20], [21], [22], [23]]. Two-group studies without a control group cannot adequately account for maturation bias, thus reducing the validity of such studies [24]. Additionally, due to the shortage of nurses in Iranian healthcare centers and the lack of sufficient time for patient interaction and education [25], utilizing peer educators trained by nurses can serve as an alternative method for patient education [26].

Given the chronic nature of MS and the valuable experiences patients gain over time in managing the disease, it is essential to leverage the presence of these patients in MS societies. Utilizing the capabilities of experienced patients as a "peer group" can empower other patients. Therefore, this study aims to compare the effects of peer education versus nurse education on the quality of life and resilience of patients with multiple sclerosis.

## 2 Materials and methods

### 2.1 Study design and participants

This study was a randomized clinical trial with three groups, conducted at the Multiple Sclerosis Society Center of Hamadan, located in northwest Iran, from March 2023 to January 2024. The research population comprised all patients with multiple sclerosis covered by the Hamadan MS Society.

### 2.2 Sample size and calculation method

Based on similar studies [22]and considering a 95 % confidence level (Type I error of 5 %), a test power of 90 %, and accounting for a potential 10 % dropout rate, the sample size for each group was estimated to be 40 individuals.

$$n=\frac{\left(Z_{1-\frac{\alpha }{2}}+Z_{1-\beta }\right)\left(\sigma_{1}^{2}-\sigma_{2}^{2}\right)}{{\left(\mu_{1}-\mu_{2}\right)}^{2}}$$

### 2.3 Inclusion and exclusion criteria

The inclusion criteria for the study included an age range of 20–45 years, an Expanded Disability Status score between 0 and 5.5, the ability to communicate, read, and write, at least 6 months since diagnosis, and a relapsing-remitting disease course. Exclusion criteria included absence from more than one session of the educational program, hospitalization during the intervention, participant withdrawal of consent to continue in the study, and changes in medication used for MS treatment.

### 2.4 Sampling method

**Fig. 1:**
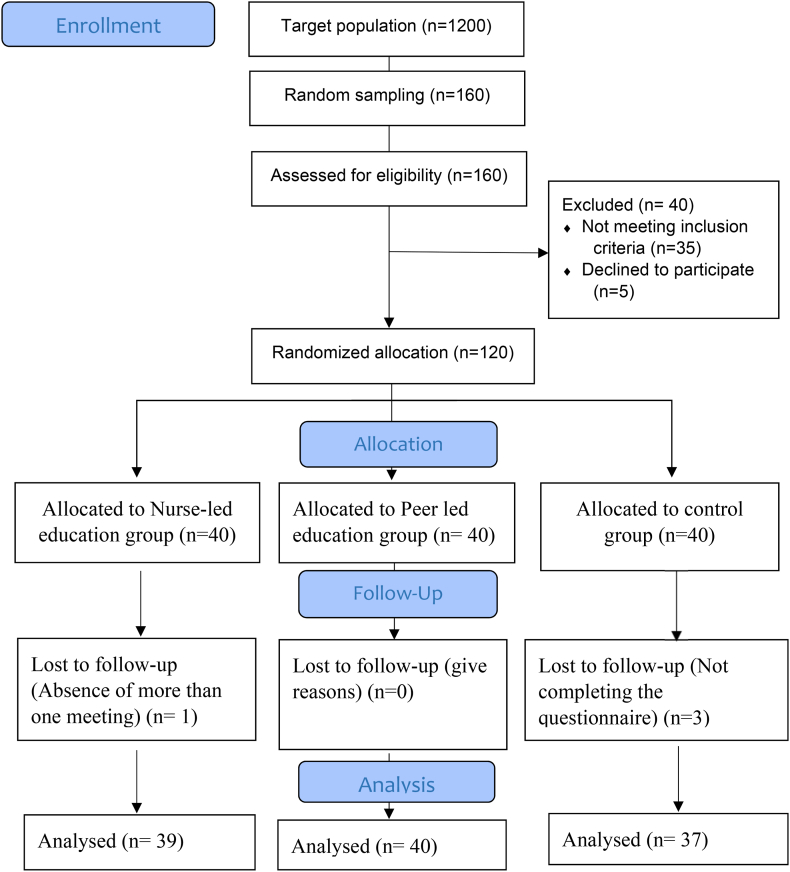
CONSORT Flowchart of study.

The sampling method used was simple random sampling. Initially, a list of patients with multiple sclerosis was prepared alphabetically. Out of 1200 patients from this society, 160 were randomly selected using a random number table. These patients were assessed to determine if they met the inclusion criteria. Forty individuals were excluded due to not meeting the inclusion criteria or unwillingness to participate in the study. The remaining 120 patients were then randomly assigned to three groups using sealed cards (Fig. 1).

Initially, 120 cards were prepared, with the letter C (control group) on 40 of them, the letter P (peer education group) on another 40, and the letter N (nurse education group) on the remaining 40. These cards were placed inside a sealed envelope. The selected samples were asked to randomly choose one card without replacement, and they were then assigned to the group indicated on the card inside the envelope.

### 2.5 Measurements

Data collection tools in this study included a form for demographic and clinical information of patients, the Connor-Davidson Resilience Scale (CD-RIS), and the multiple sclerosis quality of life (MSQOL) questionnaire.

#### 2.5.1 Demographic and clinical information form of patients

The demographic information form included personal and clinical details of patients such as age, age at onset, gender, marital status, household income, education, number of relapses in the past year, type of medication used, and insurance coverage.

#### 2.5.2 Connor-Davidson Resilience Scale (CD-RIS)

The Connor-Davidson Resilience Scale (CD-RISC) (2003) consists of 25 items rated on a Likert scale from zero (not true at all) to four (true nearly all the time), with a minimum possible score of zero and a maximum of 100. A higher score indicates greater patient resilience. This scale includes five dimensions: Perception of Personal Competence (8 items), Trust in Individual Instincts and Tolerance of Negative Affect (7 items), Positive Acceptance of Change and Secure Relationships (5 items), Control (3 items), and Spiritual Influences (2 items). A score of 50 or above suggests high resilience, while scores below 50 may indicate lower resilience [27]. In the research by Bakhshayesh Eghbali et al. experts confirmed the validity of the questionnaire. The reliability of the questionnaire was calculated using Cronbach's alpha at 0.91 [28]. In the current study, the Cronbach's alpha of the questionnaire was estimated at 0.89.

#### 2.5.3 Multiple sclerosis quality of life (MSQOL) questionnaire

The MSQOL questionnaire consists of 29 questions, with the first 20 assessing the physical impact and the last 9 assessing the psychological impact of MS on the patient. The questions are rated on a 5-point Likert scale, with scores ranging from 1 to 5. Hassanzadeh et al. translated and localized the MSQOL-29 into Persian. The minimum possible score is 29, and the maximum is 145. Scores between 29 and 58 indicate a low quality of life, scores between 58 and 87 indicate a moderate quality of life, and scores above 87 indicate a high quality of life in MS patients [29]. The Cronbach's alpha coefficients for the physical and psychological scales were 0.7 and 0.9, respectively, which are considered acceptable [29]. In this study, the reliability of this questionnaire was calculated using the test-retest method with r = 0.89.

### 2.6 Data collection

Once the research participants met the inclusion criteria, they independently completed the questionnaires in a class available at the MS Society. To ensure accurate responses, participants were encouraged to complete the questionnaires calmly and attentively. Following this, they were divided into three groups. To prevent the sharing of information, educational sessions for the two groups were scheduled on separate days. Efforts were made through careful planning and coordination with the MS Society to prevent communication among the patients during the study. Participants were asked not to share any information with other patients until the study concluded.

#### 2.6.1 Peer selection and preparation

The selection of a peer individual commenced by identifying a suitable candidate. During this phase, the researcher selected an individual with MS who had a minimum of five years of experience living with the condition. Criteria for selection included demonstrated adaptability in managing the disease, strong social connections, ability to facilitate sessions, teaching skills, and fewer disease symptoms verified by the society's physician. The chosen peer also held a bachelor's degree in architecture. To prepare this peer educator, they underwent four 1-h training sessions conducted by the research team's physician and principal investigator. Following the training, the peer's competency to educate patients was validated by a neurology specialist.

#### 2.6.2 Peer education group intervention

In this group, instruction was conducted by a trained peer over the course of five sessions, each lasting between 45 and 60 min, held every two days in a classroom at the MS Society. The peer group education technique originates from Bandura's social learning theory, which posits that individuals learn through observation, imitation, and modeling from one another. The goal of this type of education is to cultivate knowledge, attitudes, and healthy hygiene behaviors among individuals who are not specifically trained in the subject but have shared experiences [18]. The group was divided into two subgroups, each comprising 20 participants, with separate sessions organized for each subgroup. The peer employed a comprehensive approach, incorporating lectures, interactive question-and-answer sessions, and the sharing of personal experiences to educate the patients. The curriculum covered critical topics such as understanding the disease, its symptoms and complications, management strategies, adherence to treatment, mitigation of complications, medication knowledge, self-care, fostering hope, stress management, the importance of social connections, leisure, and rest. Furthermore, the peer fostered a supportive environment by sharing their own experiences with the patients, providing emotional and psychological encouragement, and addressing any inquiries or concerns raised by the participants at the conclusion of each session.

#### 2.6.3 Nurse education group intervention

Initially, participants in this group were divided into two subgroups of 20 to facilitate better instruction, with sessions held separately for each subgroup. In the nurse-led education group, the training was provided by the principal investigator, a nurse with five years of experience. The instruction in this group was also delivered over five sessions, each lasting 45–60 min, held every two days in a classroom at the MS Society of Hamedan. The nurse utilized various teaching methods, including lectures, question-and-answer, and discussions about patients' experiences. The educational content precisely mirrored that of the peer group. Additionally, the nurse addressed the patients' questions at the end of each session.

#### 2.6.4 Control group

This group did not receive any educational intervention during the study period.

The educational intervention in both groups lasted for 15 days. Two months after the completion of the intervention, the MSQOL and Connor-Davidson Resilience questionnaires were self-reported by the patients at the MS Society's location. The entire study period lasted 75 days.

### 2.7 Ethical considerations

This study received approval from the Ethics Committee of Hamadan University of Medical Sciences under the registration number IR. UMSHA. REC. 1401. 979 and was registered in the Iranian Registry of Clinical Trials on March 08, 2023 with the code IRCT20120215009014N464. Prior to participation, all objectives of the study were thoroughly explained to the participants, and written informed consent was obtained. Additionally, following the conclusion of the intervention, participants were provided with an educational booklet.

### 2.8 Data analysis

Data analysis was performed using SPSS version 16 software. The normality of the data was assessed using the Kolmogorov-Smirnov test. Subsequently, a series of statistical tests were employed, including the chi-square test, Fisher's exact test, paired *t*-test, one-way analysis of variance (ANOVA), and Bonferroni post-hoc test. A significance level of less than 0.05 was deemed statistically significant.

## 3 Results

Initially, 120 individuals were enrolled in the study, from which four were subsequently excluded, resulting in the final analysis comprising 39 individuals in the nurse education group, 40 in the peer education group, and 37 in the control group (Fig. 1). The study data exhibited a normal distribution (p > 0.05), thus allowing for the application of parametric tests for data analysis.

The results of the one-way analysis of variance did not reveal any statistically significant differences in age, age of onset of MS, or the average Expanded Disability Status Score among the three groups: nurse education, peer education, and control (p > 0.05). Furthermore, the outcomes of the chi-square test and Fisher's exact test indicated no statistically significant disparities between the groups in terms of gender, number of relapses in the past year, household income, marital status, and level of education (p > 0.05). Predominantly, most patients across all three groups were female, experienced no relapses in the past year, had a moderate household income, held a diploma-level education, and were married (Table 1). Additionally, the groups were compared regarding medication usage, revealing no significant distinctions; the majority of patients in all three groups were using Rituximab (p > 0.05), and all participants had health insurance.

**Table 1:** Comparison of demographic and clinical variables among the three study groups.

Variables		Nurse Group (n = 39)	Peer group (n = 40)	Control group (n = 37)	P-value
		M±SD	M±SD	M±SD	
Age (year)		36.02 ± 7.40	37.55 ± 5.50	35.81 ± 6.40	a0.43
Age of onset of disease(year)		25.74 ± 7.46	28.25 ± 8.06	27.65 ± 6.38	0.29^a^
The expanded disability status scale		2.31 ± 1.02	2.53 ± 1.30	2.24 ± 1.21	0.54^a^

Variables		**Frequency (%)**	**Frequency (%)**	**Frequency (%)**	P-value

Gender	Male	5 (12.80)	6 (15.00)	6 (16.20)	0.95^b^
	Female	34 (87.20)	34 (85.00)	31 (83.80)	
The number of relapses in the past year	No recurrence	29 (74.36)	26 (65.00)	27 (73.00)	c0.82
	Once	6 (15.38)	11 (27.50)	7 (18.90)	
	Twice	3 (7.70)	2 (5.00)	3 (8.10)	
	More than twice	1 (2.56)	1 (2.50)	0 (0.00)	
Household income	Enough	8 (20.51)	7 (17.50)	6 (16.20)	0.40^b^
	Medium	21(53.85)	29 (72.50)	23 (62.20)	
	Insufficient	10 (25.64)	4 (10.00)	8 (21.60)	
Marital status	Married	27 (69.20)	27 (67.50)	22 (59.50)	0.47^c^
	Single	8 (20.50)	12(30.00)	14 (37.80)	
	Isolated	3 (7.70)	1 (2.50)	1 (2.70)	
	Deceased wife	1 (2.60)	0 (0.00)	0 (0.00)	
Level of education	Literacy for reading and writing	4(10.26)	5 (12.50)	0 (0.00)	c0.14
	Cycle	3 (7.69)	7 (17.50)	6 (16.22)	
	Diploma	14 (35.90)	15(37.5)	10 (27.03)	
	Bachelor's degree	13 (33.33)	6 (15.00)	13 (35.14)	
	Above bachelor's degree	5 (12.82)	7 (17.50)	8 (21.62)	

a ANOVA.

b Chi square.

c Fisher Exact Test.

The results of the one-way analysis of variance test showed no statistically significant differences in the mean resilience and its dimensions, including personal competence, trust in one's instincts and tolerance of negative affect, positive acceptance of change and secure relationships, control, and spiritual influences, as well as the quality of life and its physical and psychological dimensions, among the three groups (nurse-led education, peer-led education, and control) before the intervention (p > 0.05). However, these differences became statistically significant in the total resilience score and its dimensions, as well as the quality of life and its dimensions, post-intervention (p < 0.05). To follow up on the differences between the groups post-intervention, the bonferroni post hoc test was used, and the results are presented in Table 3. Additionally, the paired *t*-test results showed a statistically significant increase in the mean scores of resilience and all its dimensions, as well as the quality of life and its dimensions, in the nurse-led and peer-led education groups post-intervention compared to pre-intervention (p < 0.05). However, these differences were not statistically significant in the control group (p > 0.05) (Table 2).

**Table 2:** Comparison of the mean quality of life, resilience, and their dimensions of patients with multiple sclerosis before and after the intervention between and within the three groups.

Variables	Time of evaluation	Nurse-led education (n = 39)		Peer-led education (n = 40)		Control (n = 37)		F	P value^a^
		Mean	SD	Mean	SD	Mean	SD		
Personal competence (0–32)	Before	16.56	2.74	17.40	3.86	18.03	2.69	2.055	0.133
	After	20.92	2.58	20.95	3.56	18.19	2.44	11.192	<0.001
	T	−9.43		6.35		−0.54			
	P value^b^	< 0.001		< 0.001		0.59			
Tolerance of negative affect (0–28)	Before	13.28	2.11	12.73	2.35	12.11	2.08	2.73	0.07
	After	16.46	3.45	15.70	2.65	12.19	2.31	15.85	<0.001
	T	−8.15		−9.56		−0.21			
	P value^b^	< 0.001		< 0.001		0.84			
Positive acceptance of change (0–20)	Before	10.10	20.04	9.58	2.05	90.08	1.64	2.68	0.073
	After	13.18	2.11	12.33	1.89	9.22	1.55	47.02	<0.001
	T	−16.62		−27.59		−1.30			
	P value^b^	< 0.001		< 0.001		0.20			
Control (0–12)	Before	6.10	1.31	5.80	1.02	5.51	0.84	2.84	0.06
	After	6.97	1.33	6.60	1.15	5.73	0.93	11.62	<0.001
	T	−4.25		−4.00		−1.85			
	P value^b^	< 0.001		< 0.001		0.07			
Spiritual influences (0–8)	Before	4.59	1.02	4.10	1.01	4.35	0.79	2.64	0.08
	After	5.13	1.03	5.50	1.09	4.24	0.86	15.85	<0.001
	T	−2.94		−6.63		1.00			
	P value^b^	0.01		< 0.001		0.32			
Total resilience (0–100)	Before	50.64	4.62	49.60	5.97	49.08	4.07	0.97	0.381
	After	62.67	5.63	61.08	5.42	49.57	4.20	72.99	<0.001
	T	−14.55		−15.07		−0.86			
	P value^b^	< 0.001		< 0.001		0.40			
Physical quality of Life (20–100)	Before	74.82	16.19	74.48	13.50	71.46	15.99	0.555	0.58
	After	83.46	10.75	79.33	15.16	70.86	15.25	8.107	0.001
	T	−3.37		−2.16		0.85			
	P value^b^	0.002		0.04		0.40			
Psychological quality of Life (9–45)	Before	27.59	8.96	29.85	13.71	27.57	8.28	0.594	0.554
	After	33.69	7.64	33.60	8.39	28.78	6.92	5.034	0.008
	T	−4.12		−1.64		−1.52			
	P value^b^	< 0.001		0.02		0.14			
Total quality of life (29–145)	Before	102.41	21.79	104.33	23.21	99.03	20.63	0.57	0.57
	After	117.15	16.21	112.93	20.21	99.65	19.35	90.04	<0.001
	T	−4.20		−2.42		−0.57			
	P value^b^	< 0.001		0.02		0.57			

a One way ANOVA،.

b Paired *t*-test.

**Table 3:** Pairwise comparisons of quality of life and resilience, and their dimensions post-intervention using the Bonferroni test across three groups.

Variables	Group (I)	Group (j)	Mean Difference (I-J)	Std. Error	P value^a^
Personal competence	Education by the nurse	Peer education	−0.03	0.66	1.000
		Control	2.73	0.67	<0.001
	Peer education	Control	2.76	0.66	<0.001
Tolerance of negative affect	Education by the nurse	Peer education	0.76	0.64	0.713
		Control	4.27	0.65	<0.001
	Peer education	Control	3.51	0.65	<0.001
Positive acceptance of change	Education by the nurse	Peer education	0.850	0.42	0.134
		Control	3.960	0.43	<0.001
	Peer education	Control	3.11	0.43	<0.001
Control	Education by the nurse	Peer education	0.370	0.26	0.454
		Control	1.240	0.26	<0.001
	Peer education	Control	0.87	0.26	0.004
Spiritual influences	Education by the nurse	Peer education	−0.37	0.23	0.305
		Control	0.88	0.23	0.001
	Peer education	Control	1.26	0.23	<0.001
Total resilience	Education by the nurse	Peer education	1.59	1.16	0.52
		Control	13.10	1.18	<0.001
	Peer education	Control	11.51	1.17	<0.001
Physical quality of Life	Education by the nurse	Peer education	4.14	3.120	0.563
		Control	12.60	3.180	<0.001
	Peer education	Control	8.460	3.160	0.026
Psychological quality of Life	Education by the nurse	Peer education	0.090	1.73102	1.000
		Control	4.91	1.77	0.019
	Peer education	Control	4.82	1.75	0.021
Total quality of life	Education by the nurse	Peer education	4.23	4.20	0.95
		Control	17.51	4.28	<0.001
	Peer education	Control	13.28	4.26	0.007

a Bonferroni post hoc test.

The bonferroni post hoc test was used to follow up on the differences between the groups post-intervention. The results indicated that the mean resilience and its dimensions, including personal competence, trust in one's instincts and tolerance of negative affect, positive acceptance of change and secure relationships, control, and spiritual influences, as well as the quality of life and its physical and psychological dimensions, showed a significant increase in the nurse-led education group (p < 0.05) and the peer-led education group (p < 0.05) compared to the control group. However, there were no statistically significant differences between the nurse-led education group and the peer-led education group in any of the variables or their dimensions (p > 0.05) (Table 3).

## 4 Discussion

The findings indicate that both nurse-led and peer-led education interventions significantly enhance resilience and quality of life. Post-intervention, participants in these groups demonstrated notable improvements in both resilience and its dimensions, as well as in the overall quality of life, compared to the control group. These results underscore the effectiveness of structured educational programs in fostering better psychological and life quality outcomes. This suggests that the utilization of five educational sessions in both groups effectively enhanced the resilience and quality of life among MS patients.

The improvement in quality of life and resilience, along with their dimensions, observed in peer-led groups underscores the positive impact of this educational approach. This outcome can be attributed to the social, scientific, experiential, and psychological support provided by peers, which is inherent in peer-led education. Such support has the potential to bolster patients' self-confidence. Furthermore, peer educators, by presenting successful role models, furnish patients with examples of achievement and hope, potentially bolstering their motivation. These role models may encourage patients to adhere more effectively to treatment, care, and disease management plans. Additionally, peer-led education may foster a greater sense of control over their condition among MS patients. Through the sharing of successful experiences and effective caregiving strategies, patients can enhance the skills required for disease management, mitigate disease-related complications, and alleviate the adverse effects of the disease. Consequently, the results of this study indicate that peer-led education, by providing social and informational support, presenting effective role models, and enhancing the perception of disease management capability, can facilitate the enhancement of resilience and quality of life in patients [18].

In line with our study, Jamali et al. [30]discovered that peer group education increased resilience among mothers of children with leukemia. Mollaei et al. [31] also noted significantly higher resilience levels in cancer patients within the peer group compared to the control group. Additionally, Bijani et al. [20] demonstrated that peer education enhanced the quality of life and self-efficacy in multiple sclerosis patients compared to controls. Similarly, Jahanshahi et al. [32] found that peer education effectively improved the quality of life in heart failure patients. Despite variations in sample populations, consistent findings were obtained. Moreover, Ng et al. (2013) reported improved psychological functioning and quality of life in MS patients six weeks after a peer support program compared to before the intervention [33]. Jadid-Milani et al. also demonstrated that peer-led education is effective in improving the physical quality of life for patients with MS.

However, Crotty et al. [3] found that a peer telephone support program did not effectively control pain severity in rheumatoid arthritis patients, contradicting our research findings. It could be argued that the peer education program in their study was conducted over the phone rather than face-to-face. Additionally, in contrast to our results, Uccelli et al. conducted a single-group pre-and-post study and found that 8 weeks of peer education had no impact on the quality of life and depression in MS patients, possibly due to differences in educational content, patient disability severity, and timing of assessments [22].

It appears that nurses, given their specialized field of activity, excel at presenting information in an understandable manner tailored to patients' needs. Patients perceive them as competent individuals knowledgeable about current science, serving as advisors and guides. Consequently, they trust the educational content provided by nurses and strive to apply the acquired knowledge to alleviate the complications of their disease. Aligned with the present study, a systematic review by Allen Brenner et al. (2022) summarized that nurse-led rehabilitation interventions focusing on self-efficacy and self-management significantly improved MS patients outcomes [34]. Additionally, a systematic review by Bulto et al. demonstrated that nurse-led education is effective in improving dietary adherence, increasing physical activity, reducing blood pressure, and enhancing the lifestyle of patients.

### 4.1 Peer education vs. nurse education

Interestingly, the study found no statistically significant differences between the nurse-led and peer-led education groups in terms of improvements in quality of life and resilience, along with their dimensions. This suggests that peer education, when conducted by well-trained and experienced individuals, can be as effective as traditional nurse-led education. Peer education leverages the shared experiences and empathy among patients, fostering a supportive learning environment and enhancing the educational experience. This finding is particularly relevant in contexts where there is a shortage of healthcare professionals, as it highlights the potential of peer education to fill gaps in patient education and support. A study published in the Journal of Multiple Sclerosis Care supports this, noting that MS specialist nurses play a crucial role in patient education, helping patients understand the disease process and manage their condition effectively [35]. In contrast to the present study, Dehghani demonstrated that peer-led education had a greater impact on improving health literacy among MS patients compared to lecture-based education. The differences in results could be attributed to the timing of the outcome assessment, as their study measured outcomes one month post-intervention, as well as differences in the nature of the variables being evaluated [36].

Patients also find it feasible to adhere to the nurse's advice and guidance on stress management, nutrition, physical activity, self-care, and other health-related issues. They benefit from the psychological support and sense of security provided by the nurse, thereby bolstering their resilience and quality of life. Evidently, patients place greater trust in nurses compared to peers, resulting in a notable increase in resilience and quality of life scores in the nurse-led group, although this difference did not achieve statistical significance. Moreover, Bayati et al. demonstrated that while self-care scores increased in both peer-led and nurse-led groups among hemodialysis patients, nurse-led education was more effective [37]. In our study, although the nurse-led group showed a greater increase in quality of life and resilience compared to the peer group, no statistically significant difference was observed between the two groups. This discrepancy could stem from variations in disease type, assessed variables, and the number of educational sessions.

In the control group, no significant differences were observed in the average scores of resilience and its dimensions—such as perception of personal competence, trust in individual instincts and tolerance of negative affect, positive acceptance of change and secure relationships, control, and spiritual influences—as well as in the quality of life and its physical and psychological dimensions after the intervention compared to before. This lack of change can be attributed to the absence of training during the two-month period for this group, resulting in no notable alterations in these variables. Conversely, both the nurse-led and peer-led education groups exhibited significant increases in quality of life and resilience, including their respective dimensions, after the intervention compared to before. These findings suggest that the five sessions of educational programs delivered by peers and nurses were effective in improving the outcomes in these two groups. The results underscore the importance of structured educational interventions in enhancing both psychological resilience and overall quality of life. Consistent with the present study, Mohammadpourhodki et al. demonstrated that the self-efficacy of patients in both the nurse-led and peer-led education groups significantly increased compared to before the intervention [23].

### 4.2 Implications for nursing practice

The study's findings highlight the significant benefits of integrating both nurse-led and peer-led educational interventions into clinical practice for MS patients. Nurse-led education provides specialized, evidence-based guidance, while peer-led education offers relatable support and motivation through shared experiences. Both approaches effectively enhance the quality of life and resilience of MS patients, making them valuable additions to standard care protocols.

To maximize the benefits of these educational interventions, healthcare providers should consider integrating both nurse-led and peer-led education into routine clinical practice. This could involve developing training programs for nurses and peer educators to ensure they are equipped with the necessary knowledge and skills to deliver effective education. Establishing support groups within MS societies or healthcare centers where peer-led education can take place is also essential. Implementing regular follow-up sessions to reinforce learning and provide ongoing support is crucial. Additionally, allocating resources to support the implementation and sustainability of these educational programs is necessary. By incorporating these strategies, healthcare providers can enhance the overall care and support provided to MS patients, ultimately improving their quality of life and resilience.

### 4.3 Study strengths and limitations

This study exhibits several strengths. Firstly, its triadic design sets it apart from prior research, which predominantly featured either dual-group or single-group setups. Additionally, both the sampling methodology and group allocation were conducted randomly, bolstering the study's internal validity. However, the study also presents certain weaknesses. There is a potential for information dissemination bias, and the absence of long-term follow-up to assess study outcomes is notable.

## 5 Conclusion

This study demonstrates that both nursing and peer education methods equally contribute to enhancing the resilience and quality of life of patients with multiple sclerosis (MS). Despite slight variations in the approaches, both interventions resulted in significant improvements in these key outcomes compared to the control group. The findings suggest that leveraging peer support can effectively empower MS patients, especially in settings where there is a shortage of nursing staff. Therefore, integrating peer education into MS management programs could be a valuable strategy to enhance patient well-being and resilience.

## Data availability statement

The data are available with the corresponding author and can be provided to researchers upon request.

## CRediT authorship contribution statement

**Mohammad Mehdi Siahvashi:** Writing – review & editing, Writing – original draft, Resources, Methodology, Investigation, Data curation, Conceptualization. **Morteza Shamsizadeh:** Writing – review & editing, Writing – original draft, Validation, Methodology, Investigation, Conceptualization. **Leli Tapak:** Writing – review & editing, Writing – original draft, Software, Formal analysis, Data curation. **Masoud Ghiasian:** Writing – review & editing, Writing – original draft, Validation, Methodology, Investigation, Conceptualization. **Azim Azizi:** Writing – review & editing, Writing – original draft, Validation, Supervision, Software, Methodology, Investigation, Funding acquisition, Formal analysis, Conceptualization.

## Declaration of competing interest

The authors declare that they have no known competing financial interests or personal relationships that could have appeared to influence the work reported in this paper.
